# Quantum Dots—Assisted 2D Fluorescence for Pattern Based Sensing of Amino Acids, Oligopeptides and Neurotransmitters

**DOI:** 10.3390/s19173655

**Published:** 2019-08-22

**Authors:** Marcin Zabadaj, Patrycja Ciosek-Skibińska

**Affiliations:** The Chair of Medical Biotechnology, Faculty of Chemistry, Warsaw University of Technology, Noakowskiego 3, 00-664 Warsaw, Poland

**Keywords:** pattern-based sensing, quantum dots, 2D fluorescence, neurotransmitters, oligopeptides, amino-acids, PLS-DA, EEM

## Abstract

Quantum dots (QDs) are very attractive nanomaterials for analytical chemistry, due to high photostability, large surface area featuring numerous ways of bioconjugation with biomolecules, usually high quantum yield and long decay times. Their broad absorption spectra and narrow, sharp emission spectra of size-tunable fluorescence make them ideal tools for pattern-based sensing. However, almost always they are applied for specific sensing with zero-dimensional (0D) signal reporting (only peak heights or peak shifts are considered), without taking advantage of greater amount of information hidden in 1D signal (emission spectra), or huge amount of information hidden in 2D fluorescence maps (Excitation-Emission Matrixes, EEMs). Therefore, in this work we propose opposite strategy—non-specific interactions of QDs, which are usually avoided and regarded as their disadvantage, were exploited here for 2D fluorescence fingerprinting. Analyte-specific multivariate fluorescence response of QDs is decoded with the use of Partial Least Squares—Discriminant Analysis. Even though only one type of QDs is studied, the proposed pattern-based method enables to obtain satisfactory accuracy for all studied compounds—various neurotransmitters, amino-acids and oligopeptides. This is a proof of principle of the possibility of the identification of various bioanalytes by such fluorescence fingerprinting with the use of QDs.

## 1. Introduction

Colloidal semiconductor nanocrystals—quantum dots (QDs) are one of the most significant developments in nanotechnology. With diameters in the range of 1–20 nanometers, they are constructed from elements of Group II (Zn, Cd, Hg)–VI (Se, S and Te), III–V and IV–VI of the periodic table, but until the last decade, most studies focused on II-VI QDs (CdSe or CdTe) [1]. Due to QDs small size, the electrons are confined in a limited space which leads to their unique spectral characteristics and positions the QDs properties between the properties of atoms and bulk materials [2].

With unique electro-optical properties, arising from the size-dependent and tunable photoluminescence and long-term photostability [1,3], these nanomaterials became advantageous alternatives to the commonly used molecular probes in biology and biomedical applications including bio-labelling, bio-imaging and bio-targeting [2]. Initially proposed as luminescent biological labels, they are finding new important fields of application in analytical chemistry, where their photoluminescent properties have been exploited in environmental monitoring, pharmaceutical and clinical analysis and food quality control for selective/specific detection of small molecules, ions, nucleic acids, proteins, enzymes, and other biologically important analytes [1,4,5].

Recent advances in QDs nanotechnology have slowly introduced these nanomaterials in analytical chemistry mostly as chemical sensors based on fluorescence measurements [6]; also in the form of an array for pattern-based sensing [7,8,9]. Due to the very small size and high surface-to-volume ratio their surface become of great importance [5]. Any modification of surrounding medium or interaction with chemical species, can result in significant alteration of their photoluminescent properties: emission intensity, spectral shift, or a change in the PL decay time. Their characteristic high fluorescence can be lowered (quenched) when various molecules are present in their proximity. Then energy transfer from QD to the molecule occurs that undergoes various mechanisms (FRET, PET, EE, etc.), which is observable by increase in the nonradiative decay rate of QD emission or emission at another wavelength when energy is transferred to other fluorophore. Direct quenching of fluorescence intensity can be employed for sensing, while the alternative is based on displacement assay where in contact with an analyte fluorescence is enhanced, or using QDs as reporters for other fluorescent dye for lowering of LOD [10,11]. To obtain appropriate analytical performance of QDs, various functionalization schemes were applied to QD surface [2,3,6]. Due to such high variety of cores, shells, hydrophilic coatings, functional groups [2], a great number of possible interactions with various bioanalytes resulting in changes of the optical properties of QDs is possible. Non-specific interactions (non-covalent binding) using multiple quantum dots in the form of softsensor array [12,13] could provide than characteristic, specific for a given analyte fingerprint, whose multidimensional structure could be deconvoluted with the use of numerical analysis using chemometric methods. We explore this possibility in this work, showing that using only one type of QDs it is possible to identify bioanalytes from various groups: selected neurotransmitters, amino acids and oligopeptides (di- and tripeptides). Each of this analytes influences QDs’ fluorescence characteristics, giving specific and unique fingerprint. To our best knowledge, non-specific interactions of quantum dots for multianalyte detection have not been proposed yet. Moreover, majority of analytical application of QDs considers only 0 D (zero dimensional) signal, i.e., peak height or peak shift [5,6], without taking advantage of a huge amount of information hidden in 2D fluorescence maps (Excitation-Emission Matrix, EEM, emission spectra for multiple excitation wavelengths) [4,14]. We show thereby, that 2D fluorescence assisted with non-specific interaction of QDs (QDs-2DF), can be used as an identification technique.

## 2. Materials and Methods

### 2.1. QDs and Samples Preparation

In all measurements (CdSe)ZnS QDs functionalized with carboxyl group were applied (QDCORESHELL-585-AQU, Dropsens, Llanera, Spain), which were 3.0 nm in diameter, λ_em_ = 585 nm, and used as 3.5 µM water solution. All assays were prepared in Polystyrene Immulon™ 2 HB 96-Well Microtiter EIA Plates, using 2 buffers: 10 mM phosphate buffer (PBS) pH 7.0, and 10 mM citrate buffer pH 5.0. To each well 7 µL of QDs solution was added, and filled with 200 µL of 1 mM analyte. To verify influence of QDs on identification, also samples containing only buffered analytes were considered. Controls were obtained by the addition of 200 µL pure buffer to 7 µL QDs solution. All sample types were prepared in 6 replicates.

### 2.2. D Fluorescence Measurements

**Figure 1:**
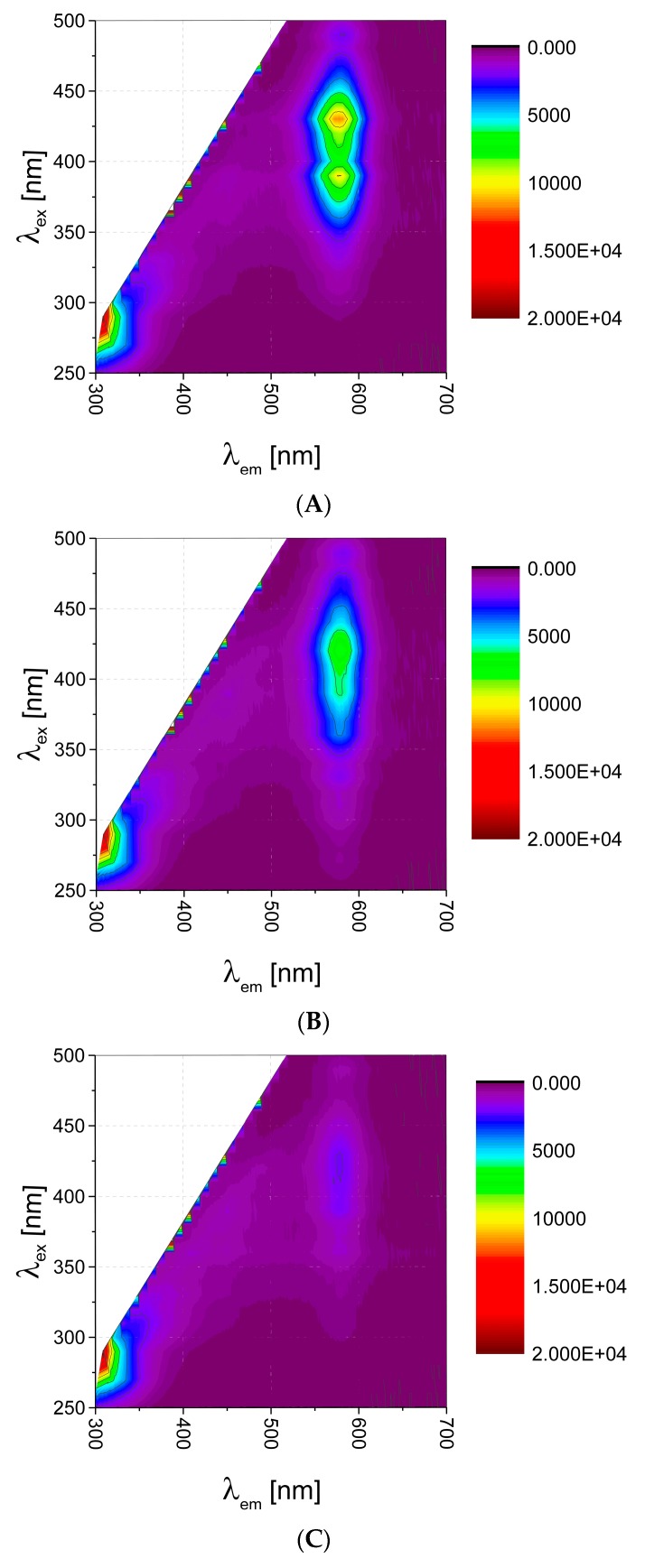
2D fluorescence map for (**A**) quantum dots λ_em_ = 585 nm, PBS pH 7.0; (**B**) quenching after addition of 1 mM Arg in PBS pH 7.0; (**C**) quenching after addition of 1 mM Gly-Ala, PBS pH 7.0.

EEMs were measured over spectra ranges of 250–500 nm (excitation) with a data interval of 10 nm and 300–700 nm (emission) with a data interval of 2 nm, at 25 °C, using Synergy 2 Multi-Mode Reader fluorescence spectrometer (BioTek Instruments, Inc., Winooski, VT, USA). It resulted in 26 × 201 data matrix per sample. Exemplary EEMs are presented in Figure 1. For further data analysis each matrix was unfolded to a vector of 3915 features (missing data, i.e., white areas on EEMs, Figure 1, were omitted). Similar strategy we applied in our previous research on 2D fluorescence [15,16].

### 2.3. Data Analysis

The chemometric analysis was performed using SOLO^®^ software (Eigenvector Research Inc., Washington, DC, USA) and Classification Learner application supplemented by in-house-written codes for Matlab (Mathworks Inc., Natick, MA, USA). Autoscalling and SIMPLS regression algorithm were applied for Partial Least Squares—Discriminant Analysis (PLS-DA). Figures, including 2D mapping, were generated using Origin (OriginLab Corporation, Northampton, MA, USA).

## 3. Results and Discussion

QDs have broad absorption spectra with narrow, sharp, symmetrical fluorescence emission peak. Studying their EEM landscape (Figure 1A), one can observe this characteristic behavior. There is peak broad in excitation mode, whereas symmetrical and quite narrow in emission mode, with maximum of 585 nm. High quantum yield (usually above 20%) can be quenched due to interaction with various biomolecules, which is facilitated by large biochemically-accessible surface area. In our case, by using carboxyl-functionalized QDs, we expected quenching by non-specific interactions with various functional groups of biomolecules (van der Waals forces, electrostatic interactions, H bonds, simple non-covalent absorption of biomolecule to QD surface [2,17,18,19], also interactions with the QD shell [6]). Such quenching effect of various magnitude was observed for various bioanalytes—exemplary EEM maps for amino acid and dipeptide presented in Figure 1 reveal, that such 2D fluorescence signature could be specific for a given analyte, and the changes on fluorescence fingerprints can be useful for bioanalytes identification.

**Figure 2:**
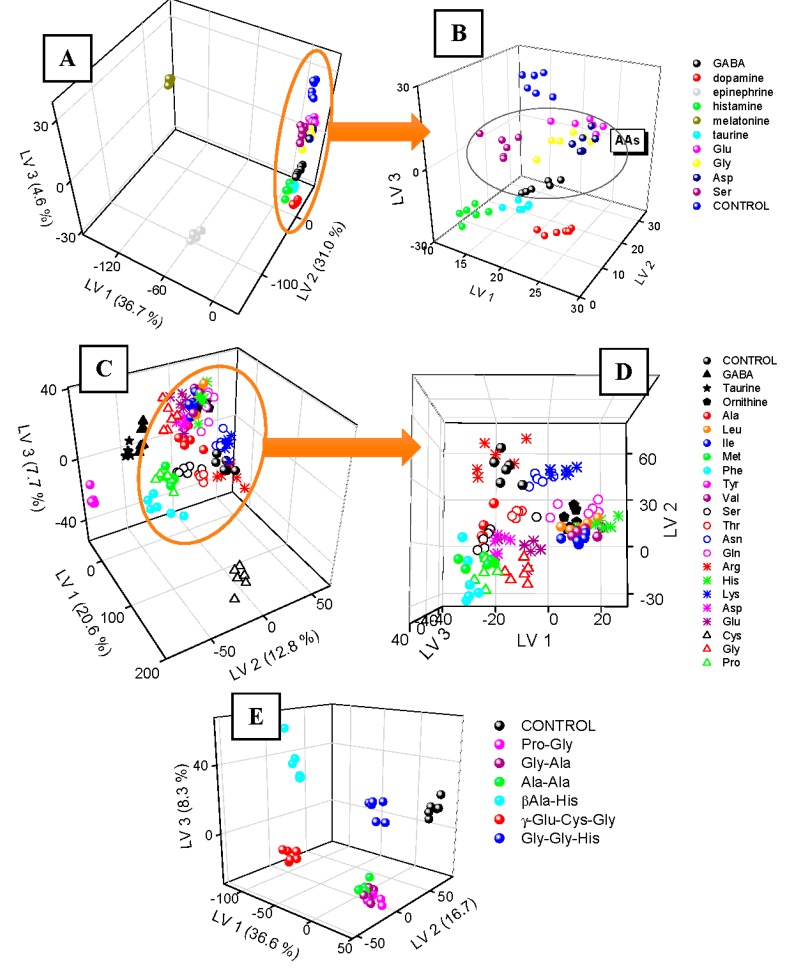
PLS-DA score plot for identification of neurotransmitters (**A**,**B**), amino acids (**C**,**D**) and oligopeptides (**E**).

To explore possible uniqueness of QDs-2DF fingerprints, 10 exemplary neurotransmitters of differentiated chemical structure and in two various pH conditions (pH 5.0 and 7.0) were considered. Their QDs-2DF maps after acquisition were processed by means of PLS-DA and presented in 3D score plot in Figure 2A. Replicates of the same type samples formed clusters, which are easily discernable from controls, i.e., pure QDs solution. Two neurotransmitters: melatonin (tryptophan derivative) and epinephrine (tyrosine derivative, catecholamine) formed distant groupings, due to their own strong autofluorescence (λ_ex_/λ_em_ 285 nm/360 nm and 280 nm/317 nm, respectively). Fluorescent dopamine (tyrosine derivative, catecholamine, λ_ex_/λ_em_ 279 nm/320 nm) is also remote from controls, and the rest analytes are placed in between dopamine and controls. Close-up of Figure 1A presented in Figure 1B gives detailed view on their fingerprints. Here, replicates are grouped together, but some of the clusters are overlapping. Nevertheless, all amino acid neurotransmitters (having both amino and carboxyl functional groups) are close to each other, closer to controls than histamine and taurine, both having amino group and without the carboxyl. Taurine, as non-proteinogenic β-amino acid with sulfonic group instead of carboxyl, is closer to the other amino acids than histamine (no acidic group). Glu, Gly, Asp formed overlapping clusters, the closest to controls. This effect can be explained by high similarity of QDs-2DF fingerprints of these amino acids to 2DF fingerprints of pure QDs. This, in turn, is possibly linked with small QDs-analyte interactions. Negatively-charged side chains of Asp and Glu are not attracted by -COOH groups of QDs, thereby their presence did not alter significantly fluorescence properties of QDs, as well as the simplest amino acid (Gly). Uncharged, but polar side chain of Ser causes slightly better discrimination from controls. All these findings show, that similarities of chemical structures can be related with changes of QDs-assisted fluorescence fingerprints. 

**Figure 3:**
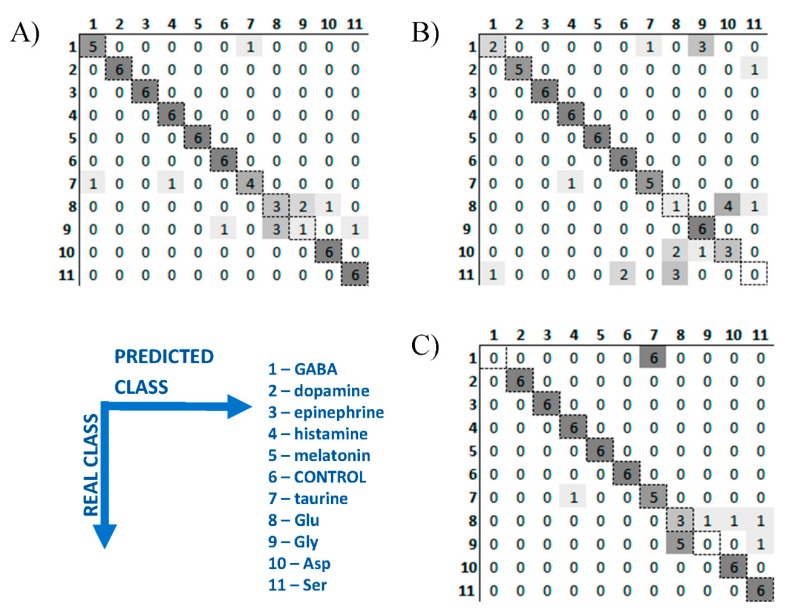
Confusion matrixes of PLS-DA models applied for neurotransmitters identification: (**A**) QDs-assisted 2D fluorescence in pH 5.0; (**B**) QDs-assisted 2D fluorescence in pH 7.0; (**C**) data fusion of (**A**,**B**).

3D PLS-DA plots for neurotransmitters transferred only 72.3% of variance of the original data, which means that the remaining almost 30% of variance still can contain information useable for the recognition of these bioanalytes. Therefore PLS-DA classification was performed with the use of cross validation and the results are presented as confusion matrix for two pH values as well as for data fusion (Figure 3A–C, respectively). It confirmed perfect identification of dopamine, epinephrine, histamine, melatonin, Asp, Ser, and controls after data fusion. The rest of neurotransmitters are classified with satisfactory accuracy, only in several cases GABA is incorrectly identified as taurine, and taurine as histamine. The most mistakes were observed for Glu, Gly and Asp at pH 7.0. This was expected looking at overlapping of their clusters in Figure 2B. The possible way of avoiding this problem and leading to better classification accuracy would be considering additional experiments at different pH value/values and/or considering more replicates which could provide more precise determination of class boundaries. Nevertheless, classification accuracy for all neurotransmitters was satisfactory (83.3%, Table 1).

The smallest discrimination among neurotransmitters was observed for amino acids, therefore more detailed study of QDs-2DF fingerprints of this particular kind of bioanalytes was performed in the next step. 22 amino acids in total were applied: 19 proteinogenic α-amino acids, 1 non-proteinogenic α-amino acid (ornithine), 1 β-amino acid with sulfonyl group (taurine), and 1 γ-amino acid (GABA). QDs-2DF fingerprints were repeatable for replicates of the same samples (Figure 2C). Distinct, easily discernable from controls clusters were formed by Tyr, Phe (both fluorescent), Cys (the only one having thiol group), GABA (the only one with amino group in γ position), and taurine (the only one with amino group in β position). The rest amino acids poses more similar chemical structure, therefore their QDs-2DF fingerprints are also more similar (Figure 2D). Even though cluster overlapping occurs, some relation between amino acid properties and their fingerprints can be noticed. Amino acids with electrically charged side chains can be easily divided into two groups: Arg, His, Lys (positive charge at side chain, LV2 > 10) and Asp, Glu (negative charge at side chain, LV2 < 10). Just between these two groups, all amino acids with polar uncharged side chain can be observed (Ser, Thr, Asn, Gln; generally higher value of LV2 than Asp and Glu and lower values of LV2 than Arg and Lys). The most overlapping cluster is formed by structurally similar Ile, Leu, Val and ornithine (all having hydrophobic side chain). It also can be seen, that distance in LV1–LV2 space between 2 amino acids differing only by one carbon in structure, i.e., Lys and ornithine, is small, which shows their very similar QDs-2DF fingerprint.

Figure 2C,D transfer only 41,1% of original variance in the data, whereas still almost 60% of information is hidden in the following latent variables, not visible on these 3D PLS plots. Therefore, even though some clusters were not ideally separated, results of classification of 22 samples are suitable. Less than 60% of accuracy was achieved for models considering fingerprints in one pH value, but data fusion achieved much better result—82.6% (Table 1). This score is high considering very similar structures of the studied 22 analytes, and possibly could be further improved by the application of non-linear classifier (there are lots of independent, uncorrelated information in the fingerprints, which is evidenced by variance loading into following latent variables).

The last step of this work was the application of the proposed method to the identification of bioanalytes more structurally sophisticated—various oligopeptides. Only non-fluorescent di- and tripeptides were applied (His fluorescence: λ_ex_/λ_em_ 220 nm/360 nm [20] is out of range of the studied EEMs). PLS score plot (Figure 2E) provided evidence for high dissimilarity of QDs-2DF fingerprints of all oligopeptides and controls. Moreover, both tripeptides formed distinct, compact clusters, as well as carnosine (β-Ala-His). The resting three dipeptides were grouped together. They change QDs fingerprint similarly, due to identical half of molecule. It again shows, that change in QDs-2DF fingerprint can be related to similarity in chemical structure of the studied analytes due to their similar interactions with QDs. It was also confirmed by looking at class affinities of the oligopeptides in the confusion matrix—only Gly-Ala was misclassified (confused with Ala-Ala and Pro-Gly), whereas all other analytes were recognized perfectly, giving total accuracy as high as 88.1% at pH 7.0 (Table 1).

## 4. Conclusions

QDs have certain advantages over conventional fluorescent dyes: high photostability, broad absorption spectra, larger biochemically-accessible surface area for bioconjugation with biomolecules, high quantum yield (>20%), and generally long fluorescence decay times (often >10 ns) [5]. Moreover, their narrow, sharp, symmetrical emission spectra and size-tunable PL enable to combine different colored QDs without spectral overlap in one assay, which is a great advantage in pattern-based sensing [2].

Nowadays, there are a lot of hydrophobic and hydrophilic QDs of different composition, size and surface functionalization available, that can be exploited for analyte recognition and/or determination. From the point of view of functionalization, carboxyl—and amine functionalized QDs are mostly offered, but aldehyde, ketone, thiol, can be also available [2]. Some of them are already conjugated with biomolecules. For aqueous solubility and opportunities for bioconjugation, QDs are coated with organic molecules and macromolecules. Such wide variety of QDs surface gives again a great advantage for pattern-based sensing, due to exploiting various interactions between various functional groups.

This work aimed to show proof of principle of the recognition and identification of various bioanalytes by fluorescence fingerprinting with the use of QDs. Although non-specific interactions of QDs are usually avoided, and regarded as their general disadvantage, we decided to avail this property for analytical purposes. The presented results of high accuracy of such classification of neurotransmitters, amino acids and oligopeptides, are only preliminary research relating chemical structure similarity to resemblance of the alteration of EEM maps of QDs. Only one type of QDs was studied, functionalized with only one type of functional group (carboxyl), giving satisfactory accuracy for all studied compounds. Further works with other types of QDs, differing by size, composition, and functionalization, are ongoing in our laboratory in order to develop QDs-based sensor arrays for higher-order sensing [4,21], which should achieve even higher accuracy of classification and give possibility of both identification and quantification of investigated analytes.

## Figures and Tables

**Table 1:** Accuracy (percent of correct classifications) of the bioanalytes identification by QD-assisted 2D fluorescence coupled with PLS-DA.

	pH 5.0	pH 7.0	Data Fusion
**10 neurotransmitters + control**	83.3%	69.7%	75.8%
**22 amino acids + control**	47.8%	57.2%	82.6%
**6 oligopeptides + control**	78.6%	88.1%	85.7%
